# From Embryo to Adult: piRNA-Mediated Silencing throughout Germline Development in *Drosophila*

**DOI:** 10.1534/g3.116.037291

**Published:** 2016-12-07

**Authors:** Pauline P. Marie, Stéphane Ronsseray, Antoine Boivin

**Affiliations:** Laboratoire de Biologie du Développement, Sorbonne Universités, Université Pierre et Marie Curie, Centre National de la Recherche Scientifique, Institut de Biologie Paris-Seine, F-75005, France

**Keywords:** piRNA, development, germline, silencing, *Drosophila*

## Abstract

In metazoan germ cells, transposable element activity is repressed by small noncoding PIWI-associated RNAs (piRNAs). Numerous studies in *Drosophila* have elucidated the mechanism of this repression in the adult germline. However, when and how transposable element repression is established during germline development has not been addressed. Here, we show that homology-dependent *trans* silencing is active in female primordial germ cells from late embryogenesis through pupal stages, and that genes related to the adult piRNA pathway are required for silencing during development. In larval gonads, we detect *rhino*-dependent piRNAs indicating *de novo* biogenesis of functional piRNAs during development. Those piRNAs exhibit the molecular signature of the “ping-pong” amplification step. Moreover, we show that Heterochromatin Protein 1a is required for the production of piRNAs coming from telomeric transposable elements. Furthermore, as in adult ovaries, incomplete, bimodal, and stochastic repression resembling variegation can occur at all developmental stages. Clonal analysis indicates that the repression status established in embryonic germ cells is maintained until the adult stage, suggesting the implication of a cellular memory mechanism. Taken together, data presented here show that piRNAs and their associated proteins are epigenetic components of a continuous repression system throughout germ cell development.

PIWI-interacting RNAs (piRNAs) are a class of single-stranded small RNAs (smRNAs), ranging from ≈23 to 29 nucleotides that ensure repression of transposable element (TE) activity in germ cells of metazoans (Aravin *et al.* 2007; Iwasaki *et al.* 2015). In *Drosophila melanogaster*, most piRNA sequences are complementary to a small number of heterochromatic genomic loci located near centromeres or telomeres. Such loci, called piRNA clusters, are heritable repositories of ancient or recent TEs (Brennecke *et al.* 2007; Senti and Brennecke 2010). In the germline, a complex made of a Heterochromatin protein 1a (HP1a) homolog, Rhino and two partners, Deadlock and Cutoff (RDC complex), ensures noncanonical dual-strand transcription of most piRNA clusters that can bypass splicing and prevent RNA pol II termination (Mohn *et al.* 2014; Zhang *et al.* 2014). Consequently, any new sequence (*e.g.*, active TE or artificial transgene) inserted into these RDC-dependent piRNA clusters will be incorporated into long chimeric piRNA precursor transcripts that further mature into piRNAs in the nuage, an optically dense cytoplasmic region surrounding nurse cell nuclei (Muerdter *et al.* 2012; Iwasaki *et al.* 2015). piRNAs are then loaded onto RNA slicing-competent Argonaute proteins [Aubergine (Aub) and Ago-3] and post-transcriptionally neutralize the expression of active TE copies through sequence complementarity. From the sliced TE mRNAs, new complementary sense piRNAs are generated that, in turn, direct cleavage of antisense precursors from piRNA clusters, a cycle termed “ping-pong amplification” (Brennecke *et al.* 2007; Gunawardane *et al.* 2007). This post-transcriptional gene silencing is reinforced by transcriptional gene silencing (TGS), mediated by nuclear Piwi-bound piRNAs and cofactors that recognize complementary nascent transcripts, thereby leading to chromatin modifications repressive for transcription (Wang and Elgin 2011; Sienski *et al.* 2012, 2015; Le Thomas *et al.* 2013; Rozhkov *et al.* 2013; Pezic *et al.* 2014; Yu *et al.* 2015).

In *Drosophila*, piRNA-mediated TE repression of female germline cells has been well- characterized in adult ovaries and ovary-derived cells, whereas very little is known about TE repression in developing germ cells. Primordial germ cells (PGCs) are the first cells to be formed in the syncytial embryo, at its posterior pole. During embryogenesis, PGCs migrate, separate into two groups, and coalesce with somatic gonadal precursor cells to form two gonads that acquire sexual identity (Dansereau and Lasko 2008). These events occur with no PGC division. After hatching of the larva, female PGCs usually undergo four nonsynchronous rounds of mitosis at different points during larval and pupal development (Gilboa and Lehmann 2006). About 10 PGCs per gonad in the late embryo give ≈150 PGCs per pupal gonad (see cycle in Supplemental Material, Figure S1). In pupal ovaries, PGCs in contact with somatic niche cells (called cap cells) remain undifferentiated and become adult germline stem cells (GSCs) (Dansereau and Lasko 2008). In the adult ovary, each GSC divides asymmetrically to produce a new GSC and a cystoblast, which undergoes four rounds of mitosis with incomplete cytokinesis to form a 16-cell germline cyst (Huynh and St Johnston 2004).

Are TEs repressed throughout *Drosophila* germ cell development and, if so, what are the functional and molecular properties of this repression? Using transgene-based assays, previous results from our laboratory indicated that repression occurs in the female third instar larval gonad (Josse *et al.* 2008). Here, we found that homology-dependent *trans* silencing is active throughout female germ cell development, from the late embryonic PGCs to the pupal PGCs. We show that 14 genes implicated in the adult piRNA pathway are also required for repression in germ cells during development. piRNAs harboring a ping-pong signature were detected in third instar larval gonads and analyses of the knockdown of zygotic *rhino* showed that maternally inherited piRNAs are not sufficient to establish complete repressing capacities. We also establish that HP1a is required for the biogenesis of piRNAs coming from natural TEs. Finally, we observed incomplete repression, resembling variegation. Clonal analyses indicated that incomplete silencing was established in the embryonic germ cells and stably maintained throughout development.

## Materials and Methods

### Drosophila stocks

Flies were raised at 25°. Stocks were obtained from the Bloomington *Drosophila* Stock Center (nos. 7373, 32180, 32249, 6420, and TRiP lines) and the Kyoto *Drosophila* Genomics and Genetic Resources (no. 123282). *BC69* bears a *P-lacZ ry+* (*P*{*A92*}) enhancer trap transgene inserted in the *vasa* gene. Homozygous *P*{*A92*} females are sterile but homozygous males are fertile.

### Immunostaining

Embryos were collected on Petri dishes 18–22 hr after egg laying (AEL). After rinsing in tap water, embryos were dechorionated in 50% bleach for 4 min and rinsed again in water. Eggs were fixed in 3.7% formaldehyde in PBS containing 0.8% Triton X-100 (PBT) for 5 min, sonicated for 2 × 7 sec at maximum intensity with a BIORUPTOR (Diagenode) with agitation between the two rounds of sonication, and kept in the fixation solution for a further 15 min. Embryos were washed for 15 min three times in PBT (0.4% Triton X-100), blocked in PBT containing 1% bovine serum albumin (PBTB) for 20 min, and incubated overnight at 4° with primary antibodies diluted in PBTB. After three 15 min washes in PBT 0.4%, embryos were incubated in PBTB with secondary antibodies for 3 hr minimum. After two 15 min washes with PBS and incubation in DAPI (Sigma) (1:1000 in PBS) for 20 min, specimens were mounted in Citifluor (Biovalley). First instar larvae were collected on Petri dishes 24–28 hr AEL and treated as embryos, except for sonication (2 × 12 sec). Second and third instar larvae were sexed and female fat bodies were hand-dissected. They were treated like embryos but without sonication, and fixation was in PBT 0.4%. Gonads were observed using a Leica TCS SP5 reverse confocal microscope. *Z*-stacks of PGC-containing gonad optical sections were acquired and analyzed using Fiji software.

### Antibodies

Primary antibodies were from Developmental Studies Hybridoma Bank (DSHB): rat anti-Vasa (1:1000), mouse anti-HP1a (1:1000), mouse anti-1B1 (1:1000), and rabbit anti-β-galactosidase (1:1000; Rockland Immunochemicals). Rabbit anti-Piwi (1:1000) and rabbit anti-Ago3 (1:1000) were a kind gift from T. Kai, rabbit anti-Aub (1:1000) and guinea pig anti-Rhino (1:500) were a kind gift from B. Theurkauf. Secondary antibodies were as follows: Alexa Fluor 633 goat anti-rat IgG (H + L) (1:1000), Alexa Fluor 647 goat anti-rat IgM (µchaine) (1:1000), Alexa Fluor 594 goat anti-rat IgM (µchaine) (1:1000), and Alexa Fluor 568 goat anti-rabbit IgG (H + L) (1:1000) from Invitrogen; Alexa Fluor 594 goat anti-rabbit IgG (H + L) (1:1000), Alexa Fluor 594 goat anti-mouse IgG (H + L) (1:1000), and Alexa Fluor 594 goat anti-guinea pig IgG (H + L) (1:1000) from Life Technologies; and GFP-Booster_Atto488 (1:1000) from Chromotek.

### smRNA extraction and deep sequencing

For each genotype, 100 third instar female larvae were hand-dissected. Fat bodies and carcasses (without the head) were recovered separately. After total RNA extraction (using TRIzol), an smRNA fraction, from 18 to 30 nt in length, was obtained by separating it on a denaturing polyacrylamide gel. This fraction was used to generate multiplexed libraries with Illumina TruSeq Small RNA Library preparation kits (RS-200-0012, RS200-0024, RS-200-036, or RS-200-048) at Fasteris (http://www.fasteris.com). A Fasteris protocol based on TruSeq, which reduces 2S RNA (30 nt) contamination in the final library, was performed. Libraries were sequenced using Illumina HiSequation 2000 and 2500. Sequence reads in fastq format were trimmed from the adapter sequence 5′-TGGAATTCTCGGGTGCCAAG-3′ and matched to the *D. melanogaster* genome release 5.49 using Bowtie (Langmead *et al.* 2009). Only 19 to 29 nt reads matching the reference sequences with 0 or 1 mismatch were retained for subsequent analysis. For global annotation of the libraries (Table S1), we used release 5.49 of FASTA reference files available in FlyBase, including transposon sequences (dmel- all-transposon_r5.49.fasta) and release 20 of miRNA sequences from miRBase (www.mirbase.org). Sequence length distributions, smRNA mapping, and smRNA overlap signatures were generated from Bowtie alignments using Python and R (www.r-project.org/) scripts, which were wrapped and run in a Galaxy instance publicly available at http://mississippi.fr. Tools and workflows used in this study may be downloaded from this Galaxy instance. For library comparisons, read counts were normalized (effective depth, Table S1) to the total number of smRNAs that matched the *D. melanogaster* genome (release 5.49) and did not correspond to abundant cellular RNAs [rRNAs, snoRNAs (collectively termed miscRNAs), or tRNAs]. Library GRH116 has the lowest effective depth and was taken as the reference to normalize the other libraries (Table S1). A second normalization factor was calculated based on miRNA quantity (Table S1). However, this normalization could not be used for carcass samples since miRNA quantity was too low in carcass samples (≈3.6%) compared to that in fat body samples (>50%) (Table S1).

For smRNA mapping (Figure 3 and Figure 4), we matched each individual RNA sequence to the *42AB* locus, the *3R* extremity (Gbrowse coordinates 3R: 32,070,000 to 32,081,331 from 6.12 release) and to the *RS3* transgene and each matched position was given a weight corresponding to the normalized occurrence of the sequence in the smRNA library. When RNA sequences matched those regions repeatedly, the weight was divided by the number of hits to these regions (multiple mapping). Distributions of piRNA overlaps (ping-pong signatures) were computed as first described in Klattenhoff *et al.* (2009) and detailed in Antoniewski (2014). Thus, for each sequencing dataset, we collected all the 23 to 28 nt RNA reads matching the *42AB* locus, the *3R* extremity, or the *RS3* transgene whose 5′ ends overlapped with another 23 to 28 nt RNA read on the opposite strand. Then, for each possible overlap of 1–28 nt, the number of read pairs was counted and represented in histogram form.

Sequence reads of different genotypes were also matched to known transposon sequences (Dmel_transposon_set_BDGP_v941). Antisense read counts from all libraries were normalized as previously, and the RPKM was calculated. Figure 4E and 4F show the results for the subset of 33 transposons representative of the three classes (exclusively somatic, soma/germline, and exclusively germline expression) described in Malone *et al.* (2009). The *x*-axis indicates the number of *w* germline knockdown (GLKD) read counts (log2, to give a representation of the amount of piRNA for a given transposon) and the *y*-axis the log2 ratio of test GLKD over control GLKD. The lower the point, the greater the effect of the test GLKD on piRNA production matching a given transposon.

### Estimation of PGC distribution probability

We calculated the probability of observing the distribution of PGCs in third instar larvae gonads by random chance, considering that the repression state of each PGC is completely independent between PGCs (under a complete plastic repression hypothesis). The mean repression fraction among considered gonads is *r* = 0.6647. The probability of observing, by random chance, seven gonads presenting a clone of eight GFP-positive, repressed PGCs and three gonads presenting a clone of eight GFP-positive, nonrepressed PGCs is *P* = (*r*^8)^7 × [(1−*r*)^8]^3 = 4.75 × 10^−22^.

### Data availability

Strains are available upon request. Small RNA sequences have been deposited at the ENA under accession number PRJEB18538.

## Results

### A transgene-based assay reveals homology-dependent repression in PGCs during development

**Figure 1:**
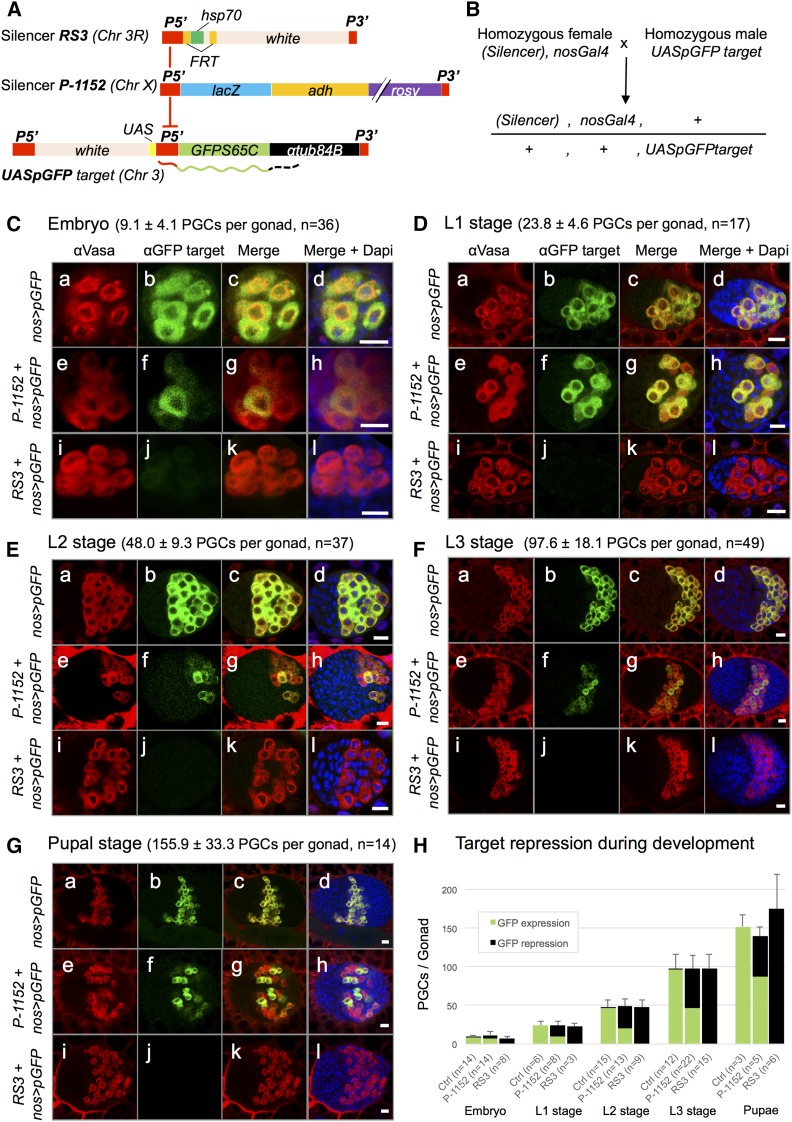
Homology-dependent repression is active during female germline development. (A) piRNA-producing transgenes (*RS3* and *P-1152* silencers) are inserted into *3R* and *X* subtelomeric regions, respectively, and share 500 bp identity with the *UASpGFP* transcripts (drawn to scale). (B) Experimental mating scheme: progeny inherit the piRNA-producing and *nosGAL4* transgenes maternally, and the *UASpGFP* target transgene, paternally. (C) Immunostainings of late embryos (18–22 hr) allow counting of PGCs (anti-VASA antibodies, first column, red) and visualization of repression (anti-GFP, second column, green). In controls (*nos > pGFP*, b), almost all PGCs express GFP, while the presence of telomeric transgenes leads to partial (with *P-1152*, f) or total (with *RS3*, j) GFP repression. Similar observations were made at the L1 (D), L2 (E), L3 (F), and pupal (G) stages. Scale bar in (C–G) corresponds to 10 µm. (H) Quantitative analysis of the immunostaining results. GFP repression is active from the late embryo and all through gonad development, partially (≈50%) with the *P-1152* silencer and completely with the *RS3* silencer. Mean number of PGCs ± SE and number of gonads analyzed (*n*) are given for each stage.

To detect homology-dependent *trans*-repression in PGCs during development, we used a transgene combination that leads to reliable and specific expression of a reporter protein in the germline. A *P{UASp-GFPS65C-αTub84B}* construct (hereafter *UASpGFP*, Figure 1A) driven by a maternally inherited *PBac{GreenEye.nosGAL4}* transgene (hereafter *nosGAL4*, Figure 1B) strongly expresses GFP in almost all germ cells from embryonic to pupal stages (99.4% GFP-positive cells, *n* = 2601, Figure 1, Cb–Gb). To test if *UASpGFP* expression could be repressed by homologous piRNAs, we used two transgenic lines sharing sequence identity with *UASpGFP* and inserted into subtelomeric regions. Strain *P-1152* contains two insertions of a *P-lacZ* construct (*P{lArB}*, Figure 1A) in a subtelomeric piRNA cluster at the *X*-chromosome tip (1A site) and produces abundant piRNAs homologous to *P*, *lacZ*, and *rosy* sequences (de Vanssay *et al.* 2012; Muerdter *et al.* 2012). piRNAs generated from telomeric *P{lArB}* copies can silence, in *trans*, other *P-lacZ* transgenes inserted into euchromatic loci, a phenomenon called *trans*-silencing effect (Roche and Rio 1998; Ronsseray *et al.* 2003; Josse *et al.* 2007, 2008). The second line, *RS3*, contains an insertion of construct *P{RS3}CB-0686-3* (Figure 1A) into a subtelomeric piRNA cluster of the *3R* chromosomal arm (100E3 site). It also produces abundant piRNAs and can silence *P-lacZ* transgenes in *trans* in adult ovaries (Dufourt *et al.* 2014; Hermant *et al.* 2015). Sequence identity between the silencer transgenes (*P-1152* and *RS3*) and the targeted transcript (*UASpGFP*) consists of a 500-bp long sequence of the *P* element (Figure 1A). Immunostainings of gonads harboring the maternally inherited telomeric silencer and *nosGAL4* transgenes, as well as the paternally inherited *UASpGFP* transgene (Figure 1B), reveal that both telomeric transgenes silenced expression of *UASpGFP* in PGCs at all developmental stages, from late embryos to pupae (Figure 1, C–G). For each gonad, we determined the number of PGCs with anti-VASA staining and the proportion of PGCs expressing GFP (Figure 1H). Repression was partial with *P-1152* and complete with *RS3* (Figure 1, C–H). Interestingly, the partial repression observed with *P-1152* resembles variegation, a stochastic bimodal repression we will address below. *RS3*-mediated repression shows a maternal effect since in reciprocal crosses, with a paternally inherited *RS3* telomeric transgene, no significant silencing was observed (94.8% GFP-positive cells, *n* = 272). This maternal effect is characteristic of the *trans*-silencing phenomenon that relies on piRNAs. Indeed, as the male gamete deposits no piRNAs, F1 females are unable to produce sufficient *de novo* transgenic piRNAs to ensure silencing (Josse *et al.* 2007; Brennecke *et al.* 2008). In conclusion, we show that a canonical *trans*-silencing phenomenon occurs in female germ cells at the embryonic, larval, and pupal stages, and that incomplete silencing can be observed as early as embryogenesis.

### Developmental silencing is sensitive to GLKD of piRNA-mediated silencing genes

**Figure 2:**
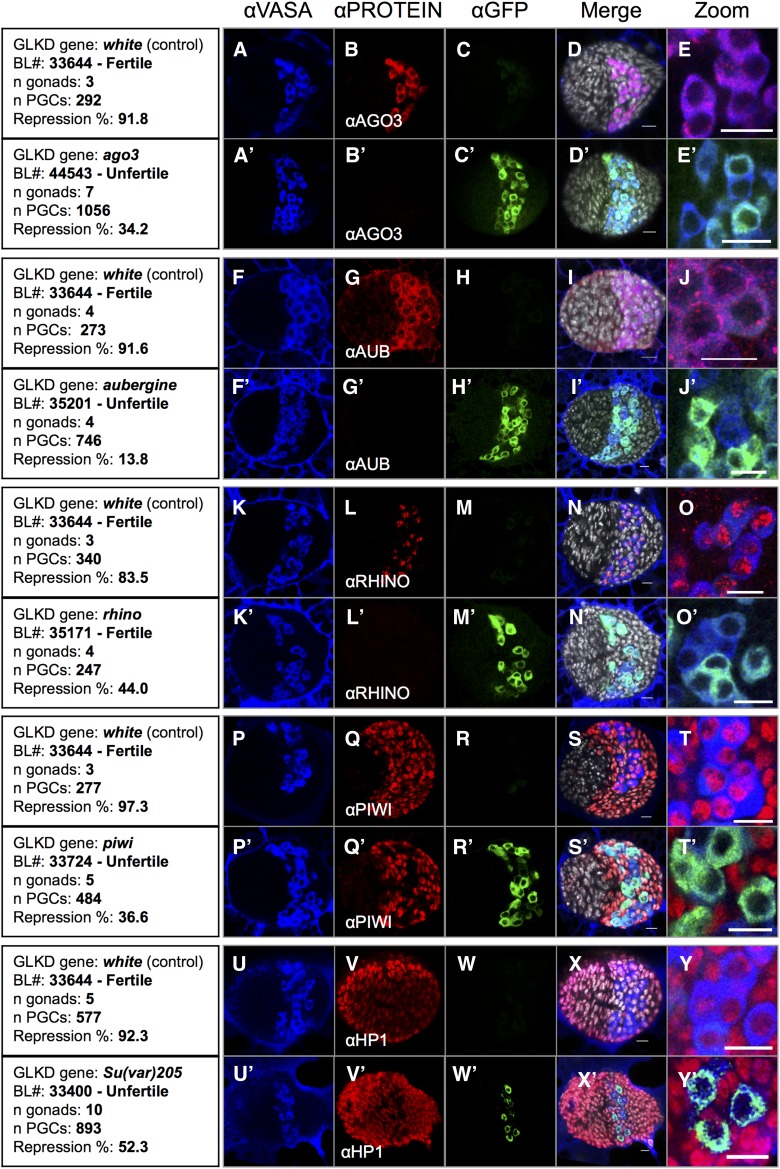
Developmental telomeric silencing is sensitive to GLKD of core partners of piRNA-mediated silencing. Immunostainings of third instar larval gonads with anti-VASA antibodies (αVASA column, blue), with anti-GFP antibodies (αGFP column, green), and with antibodies against the gene of interest (αPROTEIN column, red) are shown. Control *white* GLKD (first lane of each panel) shows strong *RS3*-induced repression of GFP expression (C, H, M, R, and W). By contrast, GLKD of core piRNA genes results in undetectable levels of the corresponding protein (B’, G’, L’, Q’, and V’), as well as presence of GFP (C’, H’, M’, R’, and W’), revealing requirement of these proteins for repression. *piwi* is also expressed in somatic cells that surround PGCs (Q and Q’). In a *piwi* GLKD context, the protein disappears specifically in the PGCs, as revealed by VASA staining (S’ and T’). Similar results were obtained with GLKD of the HP1a-encoding *Su(var)205* gene (V’, W’, X’, and Y’). DAPI is shown in gray in the merge column. Scale bar corresponds to 10 µm.

In adults, a number of genes required for piRNA-mediated silencing have been characterized [for reviews, see Iwasaki *et al.* (2015) and Hirakata and Siomi (2016)]. To test whether these genes were also required for repression throughout development of the gonad, we used the TRiP lines for expression of modified miRNA (shRNA) to knockdown specific piRNA pathway genes (Ni *et al.* 2011). In our experiments, the *nosGAL4* driver ensured shRNA-mediated GLKD. We immunostained L3 gonads carrying the maternally inherited *RS3* telomeric silencer, *nosGAL4* driver, *UASpGFP* reporter transgenes, and different paternally inherited TRiP transgenes. GFP levels indicated whether *trans*-silencing was affected upon GLKD of piRNA pathway genes. *white* GLKD gonads, in which *RS3* mediates strong GFP repression, served as negative control (Figure 2, C, H, M, R, and W). GLKD of core piRNA genes (*ago3*, *aubergine*, *rhino*, and *piwi*) resulted in disappearance of the corresponding protein, as well as expression of GFP (Figure 2, B’, C’, G’, H’, L’, M’, Q’, R’, V’, and W’). This was particularly clear for Ago3, Aub, and Rhino proteins present only in PGCs during these stages. Accumulation of Piwi was observed both in PGCs and surrounding somatic cells (Figure 2, Q and Q’). In a *piwi* GLKD context, costaining for the germline-specific Vasa protein (Figure 2, S’ and T’) confirmed that Piwi was not present in PGCs. Hence, *ago3*, *aubergine*, *rhino*, and *piwi* are required for GFP repression, probably because of their collective role in production of piRNAs. Interestingly, similar results were obtained with GLKD of *Su(var)205*, which encodes HP1a, a conserved eukaryotic chromosomal protein implicated in gene silencing through interaction with di- and tri-methylated histone three Lys9 (H3K9me2,3), also known to be implicated in transcriptional repression of active euchromatic TEs (Grewal and Jia 2007; Wang and Elgin 2011; Le Thomas *et al.* 2013; Sienski *et al.* 2015, Figure 2, V’, W’, X’, and Y’).

To avoid off-target effects and false positives, we tested, whenever possible, several different TRiP lines for inactivation of the tested gene (Table 1). The proportion of GFP-positive cells among the total number of VASA-positive PGCs was calculated for gonads of third instar larvae (Figure 2, αVASA column, and Table 1). With this quantitative approach, we can detect variation in response levels of different TRiP lines targeting a given gene (Table 1). When germline expression of at least one shRNA targeting a given gene was correlated to derepression of GFP as evidenced by immunostaining, we concluded that the function of the gene is involved in repression. However, it is not possible to conclude firmly for all the shRNAs that give negative results and for which we cannot confirm the efficiency of the shRNA due to the lack of antibodies against the products of the tested genes. We used 33 TRiP lines targeting 18 different genes. Out of 15 genes implicated in the piRNA pathway, 14 were shown to be required for repression. Hence, in addition to *ago3*, *aubergine*, *piwi*, *rhino*, and *Su(var)205*, the genes *armitage*, *cutoff*, *His2Av*, *qin*, *maelstrom*, *tejas*, *tsunagi*, *vasa*, and *zucchini* are also required to maintain repression during development (Table 1 and Figure S1). Since only one out of the three *eggless* TRiP lines weakly diminished repression, we cannot conclude as to whether *eggless* is involved in repression in female L3 gonads (Table 1). Finally, we found that GLKD of *ago1*, *ago2*, or *dicer2*, involved in miRNA and siRNA pathways, had no effect on repression (Table 1). Altogether, these results show that the main actors of all the different steps of piRNA-mediated silencing identified in adult ovaries participate in silencing during development of the germline.

**Table 1:** Quantification of repression in GLKD screen

Gene	TRiP Line Number	Fertile/Unfertile	[Gonads] PGC Number	Repressed PGCs, %
*white (control)*	33,644	F	[36] 4387	90.2
GLKD against piRNA Pathway				
*argonaute 3*	44,543	U	[7] 1056	39.1
*argonaute 3*	35,232	F	[3] 311	62.7
*armitage*	34,789	U	[6] 631	15.5
*armitage*	35,343	F	[6] 762	44.2
*aubergine*	39,026	U	[7] 924	65.8
*aubergine*	33,728	U	[11] 1847	53.3
*aubergine*	35,201	U	[4] 746	15.4
*cutoff*	35,182	F	[4] 286	51.0
*cutoff*	35,318	F	[3] 250	21.2
*eggless*	32,445	U	[11] 3502	93.7
*eggless*	34,803	F	[5] 734	94.6
*eggless*	36,797	F	[6] 1104	70.5
*His2A var*	34,844	F	[5] 493	37.7
*His2A var*	44,056	F	[7] 1094	31.4
*kumo/qin*	37,475	F	[13] 1275	49.4
*maelstrom*	34,793	U	[6] 818	21.9
*maelstrom*	35,202	U	[6] 454	32.4
*piwi*	33,724	U	[5] 484	39.9
*piwi*	37,483	F	[5] 662	86.1
*rhino*	34,071	F	[6] 662	58.0
*rhino*	35,171	F	[4] 247	39.3
*Su(var)205*	36,792	U	[7] 1333	48.6
*Su(var)205*	33,400	U	[10] 893	47.7
*tejas*	41,929	U	[10] 1098	55.8
*tejas*	36,879	U	[12] 1595	44.5
*tsunagi*	36,585	U	[5] 232	11.6
*vasa*	38,924	U	[4] 644	79.2
*vasa*	34,950	U	[5] 887	47.5
*vasa*	32,434	F	[11] 1210	45.5
*zucchini*	35,227	U	[8] 1023	68.4
*zucchini*	35,228	F	[6] 650	46.6
*zucchini*	36,742	U	[12] 1895	59.7
GLKD against miRNA or siRNA Pathways				
*ago1*	33,727	F	[6] 761	95.3
*ago1*	53,293	F	[7] 730	86.7
*ago2*	34,799	F	[8] 674	97.3
*ago2*	55,672	F	[15] 1505	88.3
*dicer2*	33,656	F	[6] 652	95.6

Quantification of target repression in GLKD shRNA screen for piRNA, miRNA, and siRNA pathway genes in third instar larvae gonads. For each considered gene are given: name in the first column, TRiP line number in the second, the fertility status in the third, the number of gonads (in brackets) and total PGCs counted per genotype in the fourth, and the percentage of repressed PGCs indicated as the number of repressed PGCs (GFP-negative cells) divided by the total number of PGCs counted (VASA-positive cells) in the last column.

### Larval PGCs contain piRNAs with a ping-pong signature

**Figure 3:**
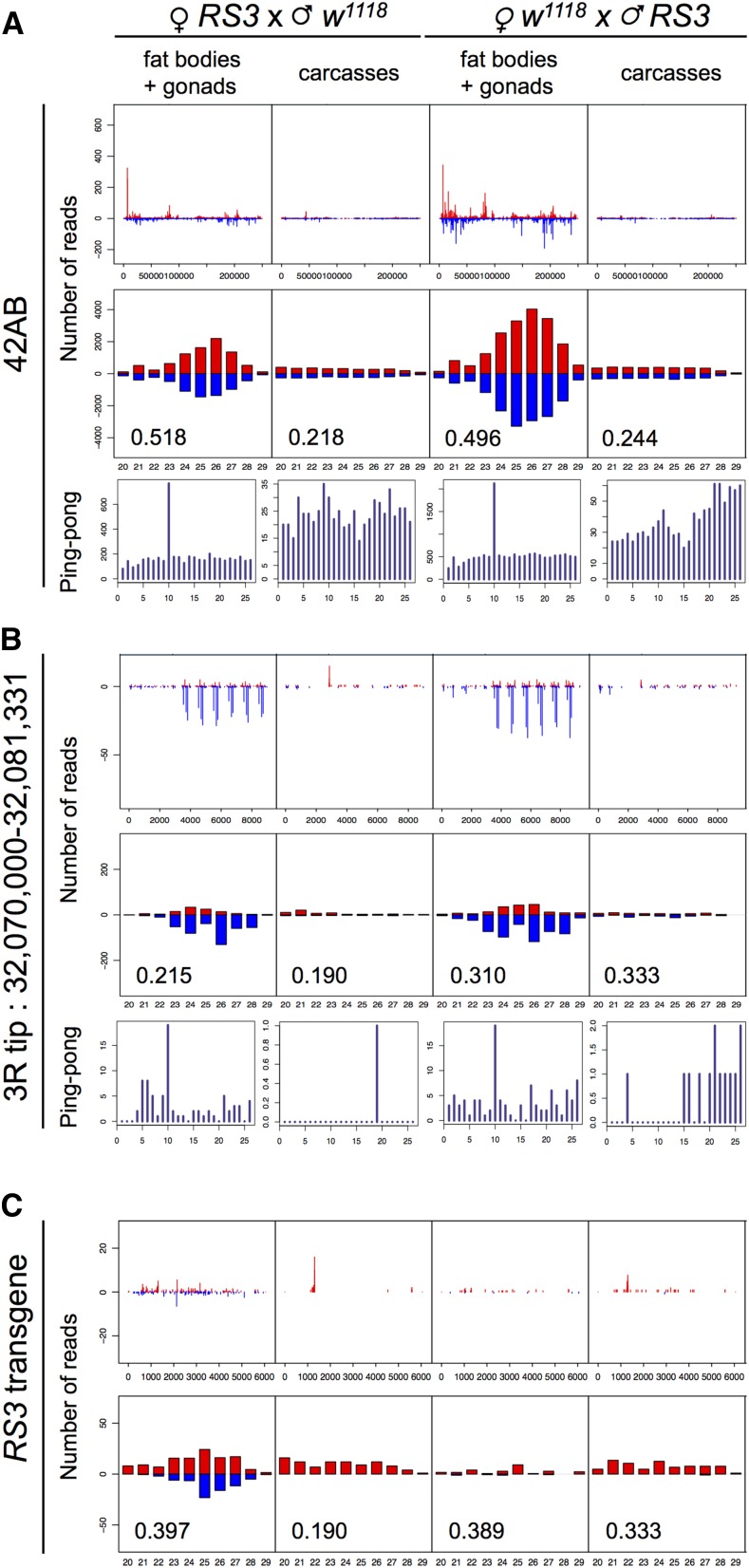
Detection of piRNAs in gonads of third instar larvae. Results of smRNA deep sequencing of gonad-containing fat bodies and carcasses of third instar larvae are presented. Parental origin of the *RS3* transgene (maternal or paternal) and tissue fraction are indicated on top. First row plots show the abundance (number of reads) of 20 to 29 nt smRNAs matching the 42AB sequence (A), the *3R* telomere (B), and the *RS3* transgene (C). Second row plots show the size distribution of 20 to 29 nt smRNAs matching the 42AB sequence (A), the *3R* telomere (B), and the *RS3* transgene (C). Positive and negative values correspond to sense (red) and antisense (blue) reads, respectively. The number in the second row of each panel is the proportion of 23–29 nt smRNAs beginning with a 5′-uridine (1U bias) (lower left corner). Third row plots (ping pong) for (A) and (B) show the number of overlapping sense–antisense smRNA pairs in the subset of 23–28 nt smRNAs (*y*-axis), as a function of the length of the overlap in nucleotides (*x*-axis). The ping-pong signature corresponds to an overlap of 10 nt between smRNAs of this size.

We looked for smRNAs in larval PGCs from reciprocal crosses between *RS3* and *w^1118^* strains. In adults, piRNA production and repression capacities of a telomeric silencer depend on maternally inherited homologous piRNAs (Josse *et al.* 2007; Brennecke *et al.* 2008). Hence, transgenic piRNAs should be detected only when the transgene is maternally inherited, whereas piRNAs produced by endogenous clusters present in all females should be detected in progeny of both crosses. Larval gonads are embedded in an organ called the fat body. PGC-containing fat bodies from ≈100 third instar female larvae were hand-dissected from carcasses, and smRNAs (smRNAs) were extracted from each tissue (heads were removed from carcasses). smRNAs from both tissues were deep-sequenced (see *Materials and Methods* and Table S1). Numerous smRNAs complementary to the 42AB region, a strong piRNA-producing locus in the adult germline, and some complementary to the *3R* telomere region, were detected in the gonad-containing fat body fraction (Figure 3, A–B). The smRNAs from both regions harbored a ping-pong signature (a high number of pairs of sense and antisense piRNAs that overlap by exactly 10 nucleotides) and presented a typical size profile from 23 to 29 nt, fully compatible with that of piRNAs (Figure 3, A–B). Interestingly, 42AB smRNAs, but not smRNAs from the *3R* telomere, presented a clear uridine bias at the 5′ end (1U bias), a piRNA characteristic (Brennecke *et al.* 2007, Figure 3, A–B). In contrast, carcass fractions did not contain significant numbers of smRNAs, and those smRNAs that were present were totally devoid of either the ping-pong signature or the 1U bias (Figure 3, A–B). Sense and antisense 23–29 nt smRNAs homologous to the *RS3* telomeric transgene were specific to progeny with maternally inherited *RS3* (RPKM value of 5.21 compared to 0.50 for paternally inherited *RS3*), fitting well with the idea that these smRNAs are indeed piRNAs (Figure 3C). They presented a strong 1U bias but not for the ping-pong signature. It is possible that the relatively low number of *RS3* reads precludes detection of the ping-pong signature.

### Larval global piRNA production depends on Rhino, while that of telomeric piRNAs depends specifically on HP1a

**Figure 4:**
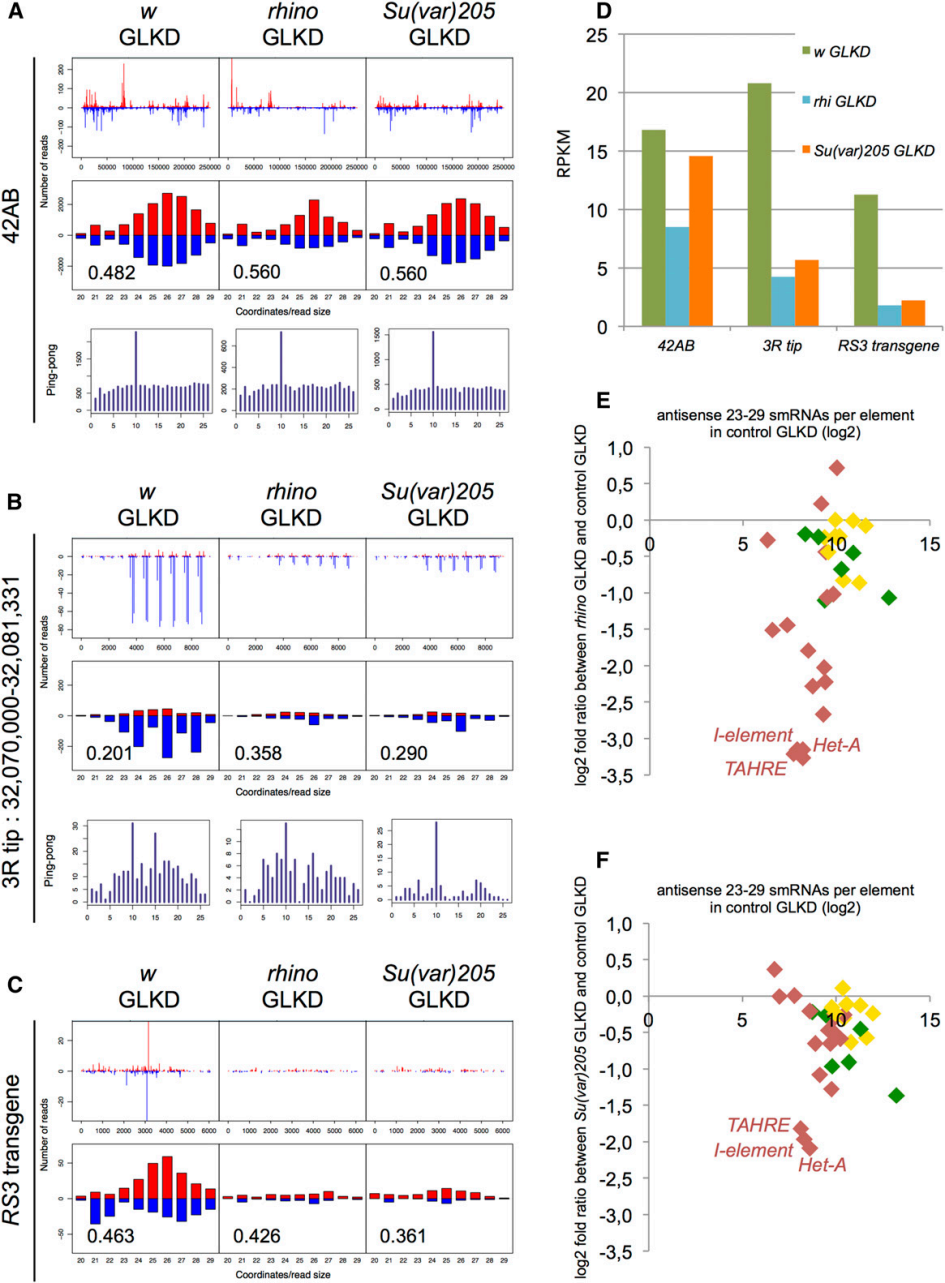
Larval piRNAs are sensitive to *rhino* GLKD and telomeric piRNAs are sensitive to S*u(var)205* GLKD. Genotypes are indicated on top. First row plots show the abundance (number of reads) of 20 to 29 nt smRNAs matching the 42AB sequence (A), the *3R* telomere (B), and the *RS3* transgene (C). Second row plots show the size distribution of 20 to 29 nt smRNAs matching the 42AB sequence (A), the *3R* telomere (B), and the *RS3* transgene (C). Positive and negative values correspond to sense (red) and antisense (blue) reads, respectively. The number in the second row of each panel is the proportion of 23–29 nt smRNAs beginning with a 5′-uridine (1U bias) (lower left corner). Third row plots (ping pong) for (A) and (B) show the number of overlapping sense–antisense smRNA pairs in the subset of 23–28 nt smRNAs (*y*-axis), as a function of the length of the overlap in nucleotides (*x*-axis). The ping-pong signature corresponds to an overlap of 10 nt between smRNAs of this size. Note that the two peaks, one sense, the other antisense, that match the *RS3* sequence near position 3000 in the *w* GLKD background correspond to *w* modified miRNAs produced by the TRiP transgene. (D) Histograms show, in RPKM, the number of reads that match *42AB*, *3R* tip, and *RS3* transgenes in control (*w*) and test [*rhino* and *Su(var)205*] GLKD contexts. (E and F) Third larval instar smRNAs corresponding to natural TEs. Scatter plots depict normalized 23–29 nt antisense smRNAs mapping to annotated TEs in test [*rhino* or *Su(var)205*] GLKD *vs.* control (*w*) GLKD log2. The *x*-axis shows the number of the *w* GLKD reads corresponding to annotated TEs (log2, to give a representation of the amount of piRNA for a given transposon) and the *y*-axis shows the log2 ratio of test [*rhino* or *Su(var)205*] GLKD over control GLKD reads corresponding to annotated TEs. The lower the ratio, the greater the GLKD effect on piRNA production matching a given transposon. *TAHRE*, *Het-A*, and *I* elements are singled out since they are affected to the greatest degree in both mutant contexts. Colors refer to classes of elements as defined in Malone *et al.* (2009): red, strong maternal deposition (germline source); yellow, intermediate maternal deposition; and green, weak maternal deposition (predominantly somatic source).

To determine whether the detected piRNAs were *rhino*-dependent and came from PGCs, we first analyzed smRNAs extracted from *rhino* GLKD larval fat body-attached gonads that had maternally inherited *RS3*. Read counts matching *42AB*, *3R* tip, and *RS3* transgene sequences in control (*w*) and test [*rhino* and *Su(var)205*] GLKD smRNA libraries were normalized using effective depth and RPKM (see *Materials and Methods* and Table S1 and Table S2). Compared to the *w* GLKD control, in which we found *bona fide* piRNAs, the *rhino* GLKD context presented a dramatic decrease in 23–29 nt smRNAs complementary to the *RS3* transgene (*w* GLKD RPKM 11.27 compared to *rhino* GLKD RPKM 1.82, Figure 4, C and D), to the *3R* subtelomeric sequences (*w* GLKD RPKM 20.82 compared to *rhino* GLKD RPKM 4.24, Figure 4, B and D), and, to a lesser decrease, the 42AB locus (*w* GLKD RPKM 16.83 compared to *rhino* GLKD RPKM 8.50, Figure 4, A and D). As *rhino* is expressed specifically in PGCs and not in somatic cells of the gonad or the fat body (Figure 2L), and as the major part of the smRNAs coming from *RS3* and the *3R* tip appear to be *rhino*-dependent, we conclude that these smRNAs likely come from PGCs. Half of the 23–29 nt smRNAs from the *42AB* region are also *rhino*-dependent and thus likely come from PGCs (Figure 4A). However, *rhino*-independent smRNAs were also detected and these could originate from germline or somatic tissues. Since these *rhino*-independent smRNAs present a strong ping-pong signature (Figure 4A), we favor the possibility that they come from PGCs. These *rhino*-independent piRNAs could also represent maternally inherited piRNAs. Taken together, our data strongly suggest that *bona fide* piRNAs are produced in third instar larval PGCs and that the loss of repression in piRNA pathway mutant contexts (Figure 2M’) is correlated to the loss of *RS3* transgenic piRNAs.

Next, we analyzed smRNAs extracted from *Su(var)205* GLKD larval fat body-attached gonads that had maternally inherited *RS3*. In this mutant context, 23–29 nt smRNAs matching the *42AB* locus appeared little affected [*w* GLKD RPKM 16.83 compared to *Su(var)205* GLKD RPKM 14.59, Figure 4, A and D], while telomeric 23–29 nt smRNAs strongly decreased [*w* GLKD RPKM 20.82 compared to *Su(var)205* GLKD RPKM 5.66 for *3R* subtelomeric sequences, Figure 4, C and D; and *w* GLKD RPKM 11.27 compared to *Su(var)205* GLKD RPKM 2.25 for *RS3*, Figure 4, B and D]. Note that using miRNA-based normalization, similar results were obtained, except that the effect of the *Su(var)205* GLKD appeared to be stronger, in particular on *42AB* 23–29 nt smRNA production [*w* GLKD RPKM 30.67 compared to *Su(var)205* GLKD RPKM 20.23, see Table S1 and Table S2]. These results indicate that there is a strong requirement for HP1a for production of telomeric piRNAs, while pericentric piRNA production relies on HP1a to a lesser degree. To date, HP1a has mainly been shown to be an effector of the piRNA pathway, required for locking target expression through TGS (Wang and Elgin 2011; Sienski *et al.* 2015). However, our previous work showed, using RNAse protection assays, that production of subtelomeric transgenic smRNAs in adult ovaries decreased upon removal of one dose of HP1a (Todeschini *et al.* 2010). Our present results provide further evidence for a role for HP1a in piRNA production. Interestingly, we found that HP1a and Rhino proteins largely colocalize in distinct zones of PGC nuclei, but localization of each protein in these nuclear zones does not depend on the presence of the other protein (Figure S2).

In order to extend the analysis to endogenous TEs, we compared the amounts of 23–29 nt antisense smRNAs matching known TEs in third instar larvae gonads from control GLKD and *rhino* GLKD contexts. By comparing ovarian and embryonic piRNAs, TE piRNAs have been classified as having germline or somatic sources (Malone *et al.* 2009). Figure 4E shows that the level of most germline piRNAs was decreased in a *rhino* GLKD context (in red in Figure 4E). By contrast, all somatic piRNAs were highly expressed in even a *rhino* GLKD context (in green and yellow in Figure 4E). As somatic piRNAs are known to be depleted in the 0–2 hr embryo (Malone *et al.* 2009), our results indicate that *de novo* somatic piRNAs are produced in large amounts sometime between embryogenesis and the L3 stage. Interestingly, when we compared amounts of 23–29 nt smRNAs matching known TEs in control GLKD and *Su(var)205* GLKD contexts, we observed that piRNA production corresponding to only three TEs depended on the presence of HP1a (Figure 4F). *HeT-A* and *TAHRE* elements constitute the ends of telomeres, while the *I* element, a recent invader of the *D. melanogaster* genome, may have different loci serving for piRNA production, including telomeres. Thus, our results indicate that HP1a is specifically required for piRNA production of at least three different telomeric sequences: *3R* subtelomeric sequences (Figure 4, B and D), as well as *HeT-A* and *TAHRE* sequences.

### Incomplete silencing reveals cellular memory from embryonic PGCs to adult GSCs

**Figure 5:**
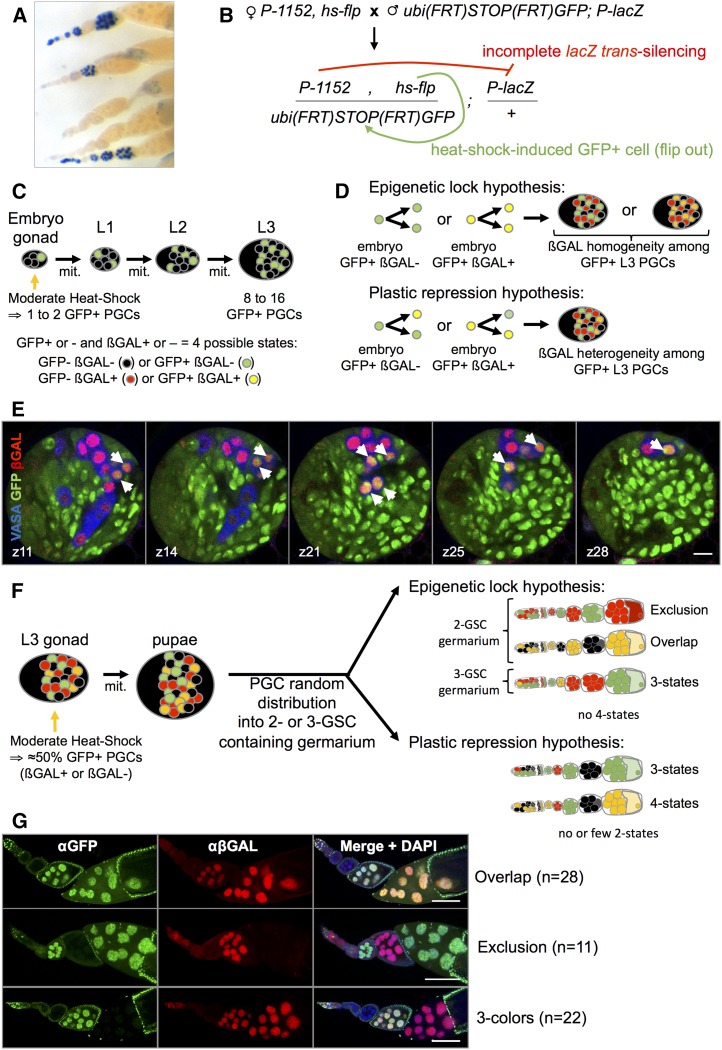
Cell lineage analysis of incomplete repression reveals cellular memory of the repression state. (A) βGAL overnight staining (dark blue) of adult ovarioles bearing the maternally inherited telomeric silencer *P-1152* and the paternally inherited euchromatic *P-lacZ* enhancer trap *BC69* reveals incomplete silencing: individual germline cysts show homogeneous ON or OFF staining. (B) Experimental mating scheme to produce progeny containing a telomeric silencer (*P-1152*), its euchromatic target (*P-lacZ*, *BC69*), a heat shock–driven flipase (*hs-flp*), and a ubiquitin promoter: GFP transgene whose expression depends on the flip-out of a *(FRT)STOP(FRT)* cassette. (C) Schematically, in a first experiment, 1–2 GFP-positive cells (in green) are induced by moderate heat-shock in 18–22 hr embryos and cell progeny observed three rounds of mitoses later in L3 gonads, giving 8–16 GFP-positive cells. The βGAL-positive or -negative status (indicated in red) is independent of GFP-positive or -negative status, thus defining four possible states that are represented by colors corresponding to immunofluorescence observations. (D) Expected results according epigenetic lock or plastic repression hypotheses are represented schematically. Under epigenetic lock hypothesis, embryonic GFP-positive PGCs keep their βGAL expression status, whether it is repressed by *P-1152* (shown in green because of the sole expression of the GFP in these cells) or unrepressed by *P-1152* [represented in yellow because of the coexpression of GFP (green) and βGAL (red) in these cells]. This leads to a homogeneous population of GFP-positive PGCs in L3, that are either all green or all yellow, depending on the βGAL status of the embryonic PGC giving rise to the clone. Under the plastic repression hypothesis, GFP-positive PGCs could change their βGAL expression status (ON>OFF or OFF>ON) during development resulting in βGAL expression heterogeneity among GFP-positive PGCs in L3. Note that GFP- PGCs appear black or red depending on the βGAL status (repressed or nonrepressed, respectively). (E) Five *z* (1 µm) confocal planes of an L3 gonad reveal that all GFP-positive PGCs are also βGAL positive (arrowheads). The *z* number indicates the focal plane (out of 31). Most somatic cells of the gonad are GFP-positive but anti-VASA staining (in blue) specifically labels PGCs. Scale bar corresponds to 10 µm. (F) Schematically, in a second experiment, GFP-positive cells were induced in late L3 and cell progeny was observed in adult ovaries. Ovarioles presenting heterogeneity for both GFP and βGAL expression were analyzed. Under the epigenetic lock hypothesis, germaria that contain two GSCs are expected to give rise to two specific phenotypes: exclusion results from one GSC GFP-positive/βGAL-negative (green) and one GSC GFP-negative/βGAL-positive (red) and overlap results from one GSC GFP-positive/βGAL-positive (yellow) and one GSC GFP-negative/βGAL-negative (black). Germariums that contain three GSC could produce three-state ovarioles. No four-state ovarioles are expected since there are never four GSCs in a germarium. Under the plastic repression hypothesis, few two-state ovarioles (exclusion or overlap phenotypes) are expected because of the plasticity of the βGAL expression status. On the contrary, ovarioles having three states and even four states should be frequently observed. (G) Distribution of phenotypes observed among 61 ovarioles analyzed by immunostaining for GFP and βGAL and nuclear staining with DAPI: examples of patterned two-state overlap and exclusion phenotypes and mixed three-state ovarioles with heterogeneous staining for both GFP and βGAL. Scale bar corresponds to 100 µm.

The first observations of incomplete piRNA-mediated silencing were made in adult ovaries (Ronsseray *et al.* 2003; Josse *et al.* 2007). Individual ovarioles presented germline cysts with full target repression and other germline cysts with no repression, and the distribution of these two types of germline cysts appeared random (Figure 5A). Nonetheless, within a given germline cyst, the status of nurse cell target repression was mostly homogenous, suggesting that this status was established at the one-cell GSC or cystoblast stage, and then maintained through the four rounds of mitosis to generate the 16-cell germline cyst. In the present analyses of piRNA-mediated repression during development, we found that incomplete silencing occurred as early as in the embryo (Figure 1Cf), and was detected at all subsequent developmental stages (Figure 1, Df–Gf). These observations raise the question of whether PGCs acquire a stable piRNA-mediated ON or OFF repression state of the target in the embryo, which is then maintained through development until the adult stage (epigenetic lock hypothesis). Alternatively, is the repression state plastic during development, changing from one state to the other, showing repression in embryos then expression at later stages, or vice versa (plastic repression hypothesis)? To discriminate between these hypotheses, we developed a genetic system to visualize the repression state of cells derived from one or two embryonic PGCs: we coupled heat shock–induced clonal cell lineage tracing, revealed by GFP expression, to *P-1152*-mediated incomplete silencing detected by β-galactosidase (βGAL) expression of a target transgene (Figure 5B). It is important to note that in this experiment, GFP expression reveals clonal lineage of PGCs and is not the reporter of *P-1152*-mediated repression—the reporter being a euchromatic *P-lacZ* transgene. Here, GFP expression is not sensitive to *P-1152*–mediated repression because of a lack of homologous sequence between *P-1152*- and *GFP*-containing transcripts. Embryos were moderately heat-shocked for 15–20 min at 37° in order to generate a low number of GFP-positive PGCs and developed at 25° until the late third larval instar. Since PGCs undergo ∼3 mitoses between embryonic and late third larval instar stages, the number of GFP-positive cells detected in late L3 allows estimation of the number of GFP-positive PGCs generated in the embryo; for example, eight GFP-positive PGCs in late L3 would derive from a flip-out event in a single PGC in the embryo (Figure 5C). The repressed or activated state of the *P-lacZ* transgene is independent of GFP clonal state. Therefore, PGCs present four possible states (Figure 5C). Under the epigenetic lock hypothesis, the repression state is expected to be homogeneous among progeny of a single embryonic GFP-positive PGC, while under the plastic repression hypothesis, it is expected to be heterogeneous (Figure 5D). Immunostainings of L3 gonads identified PGCs (VASA-positive cells), and GFP-positive cells were counted from among these. We examined 23 late L3 gonads, heat-shocked as embryos. All gonads presented incomplete βGAL repression. Among 10 gonads with eight GFP-positive cells, three presented 100% βGAL-positive staining of GFP-positive cells and seven presented 100% βGAL-negative staining of GFP-positive cells. These data strongly argue for homogeneity of repression among progeny of a single embryonic PGC. Note that under the plastic repression hypothesis, whose extreme form can be seen as the complete independence of cells for their repression state, the probability of such a distribution is *P* = 4.75 × 10^−22^ (see *Materials and Methods*). Thirteen other gonads had more than eight but <17 GFP-positive PGCs, thus likely deriving from flip-out events in two embryonic PGCs. A total of 84.6% (11 of 13) of these showed βGAL staining compatible with repression homogeneity: two had 100% βGAL-positive staining among GFP-positive cells (an example is shown in Figure 5E), four had 100% βGAL-negative staining among GFP-positive cells, and five had 50:50. Of the two last gonads, one had five βGAL-positive cells and nine βGAL-negative cells, and the other had two βGAL-positive cells and eight βGAL-negative cells. Flip-out events in three embryonic PGCs might sometimes give rise to <17 PGCs in late L3, thus explaining the first distribution, and some PGCs might divide at a lower frequency, potentially explaining the second distribution. Alternatively, the repression state could be plastic in these two gonads. Taken together, 91.3% (21 of 23) of the L3 gonads showed βGAL repression reporter activity that is fully compatible with repression homogeneity among cells derived from a single embryonic PGC, thereby supporting the epigenetic lock hypothesis.

Next, we asked whether epigenetic lock of germline piRNA-mediated repression also exists between the third instar larval and adult stages. To test this, late third instar larvae were heat-shocked for 15 to 20 min at 37°, which is long enough to induce flip-out events in ∼50% of PGCs, and were left to develop at 25° until eclosion (Figure 5F). Each ovariole contains two or three GSCs in its anterior-most structure, called the germarium (Wieschaus and Szabad 1979), and the proportion of germaria containing two *vs.* three GSCs varies from 20 to 50% (Wieschaus and Szabad 1979; Margolis and Spradling 1995). We used Hts immunofluorescence that specifically labels the spectrosome, a dotted structure present in GSCs and cystoblasts, and estimated that the proportion of two GSC-containing germaria was ∼50% in our experimental conditions (*n* > 100). Thus, about half of the ovarioles we tested had germline cysts derived from only two GSCs. Under the plastic repression hypothesis, no correlation is expected between heat shock–induced GFP-positive cells and βGAL silencing: ovarioles should contain mixed egg chambers presenting all possible combinations of GFP-positive or -negative and βGAL-positive or -negative expression. In other words, the number of different egg chamber states should exceed GSC number, *i.e.*, we should observe >50% of ovarioles with three states (Figure 5F). On the contrary, under the epigenetic lock hypothesis (*i.e.*, if L3 PGCs maintain and transmit their repression state until adults), we expect specific patterns of heat shock–induced GFP-positive cells in ovarioles with heterogeneity for both GFP and βGAL expression. First, GFP/βGAL staining exclusion is expected if one of the GSCs is positive for GFP and negative for βGAL while the other GSC is negative for GFP and positive for βGAL. Alternatively, GFP/βGAL staining overlap is expected if one PGC is positive for both GFP and βGAL and the other one is negative for both (Figure 5F). We focused on 61 ovarioles that presented with simultaneous heterogeneity for GFP and βGAL staining. More than the expected 50% of two GSC-containing ovarioles presented with specific patterns (63.9%, overlap *n* = 28 and exclusion *n* = 11) (Figure 5G). This result supports the epigenetic lock hypothesis. The remaining ovarioles presented with mixed patterns (36.1%, *n* = 22). The mixed pattern ovarioles could derive from plastic repression but we favor the hypothesis that they reflect the high proportion of germaria with three GSCs (∼50%) that could be heterogeneous for βGAL and GFP status. Moreover, under the plastic repression hypothesis, ovarioles with four different GFP/βGAL status combinations should occur but were never observed. Taken together, these studies performed from embryo to L3 and from L3 to adults suggest strongly that the repression state is maintained within a cell line between these stages, suggesting that a cellular memory mechanism must be operating through germ cell divisions.

## Discussion

Here, we investigated piRNA-mediated repression throughout female germline development, from embryonic to pupal PGCs in *Drosophila*. Using transgenes inserted into telomeric piRNA clusters, we observed silencing *in trans* of partially homologous reporter transgenes located elsewhere in the genome. We show that typical piRNA pathway genes are required for reporter gene repression in larval PGCs, while those for the siRNA or miRNA pathways are not. In addition, smRNAs corresponding to piRNAs are likely present in larval gonads. In the GLKD experiments we presented here, expression of a modified miRNA (shRNA) designed to knockdown the gene of interest depends on a paternally inherited *nosGAL4* transgene. Thus, the knockdown is zygotically induced. The fact that accumulation of some of the larval transgenic piRNAs was reduced upon zygotical GLKD of *rhino*, a gene required for the production of primary piRNAs, strongly suggests that these piRNAs are zygotic piRNAs. By extension, we propose that these piRNAs, which were found to be sensitive to *rhino* zygotic GLKD, must be piRNAs that are produced *de novo*. By contrast, piRNAs whose accumulation was insensitive to zygotic GLKD of *rhino* could be of maternal or zygotic origin. Nevertheless, the fact that under conditions inducing zygotic GLKD of *rhino*, target repression is abolished, demonstrates that maternally inherited piRNAs are not sufficient to establish complete repression in L3 PGCs.

Interestingly, *His2Av* and *Su(var)205* GLKDs exhibited strong derepression of the GFP target transgene in L3 PGCs (Figure 2 and Table 1). HP1a’s role in telomere capping (Fanti *et al.* 1998) and/or its presence on subtelomeric regions (Frydrychova *et al.* 2008) may possibly be necessary for the ability of telomeric silencers to produce piRNAs. In support of this, our previous work showed that reducing the dose of HP1a by half, abolished *P* element repression capacities mediated by *P* copies inserted in subtelomeric heterochromatin (Ronsseray *et al.* 1996). We further showed, using RNAse protection assays, that production of subtelomeric transgenic smRNAs in adult ovaries was reduced upon reduction by half of the dose HP1a (Todeschini *et al.* 2010). Here, we show that HP1a is required to produce several different kinds of telomeric piRNAs, and apparently not centromeric piRNAs, in L3 PGCs (Figure 4, C and E). However, we cannot exclude that HP1a could also be acting at the level of TGS of the *UASpGFP* target, as described for endogenous TEs in adults (Wang and Elgin 2011; Le Thomas *et al.* 2013; Sienski *et al.* 2015). Immunostaining of polytene chromosomes of third instar larvae reveal that His2Av localized at telomere tips in somatic cells (van Daal and Elgin 1992; Rong 2008). Mutations of *His2Av* suppress position effect variegation (PEV), a phenomenon that occurs when a euchromatic sequence is relocated next to heterochromatic regions (Swaminathan *et al.* 2005; Elgin and Reuter 2013). The expression of relocated genes is then subject to stochastic and bimodal (ON/OFF) expression due to the extension of heterochromatin proteins, such as HP1a, over flanking sequences to varying degrees from one cell to another. This cell-autonomous phenomenon thus produces a variegated phenotype. Mutations of *His2Av* also reduce repressive chromatin marks, such as H3K9me3 or H4K12Ac, and reduce HP1a recruitment to centromeric regions (Swaminathan *et al.* 2005). As for HP1a, we propose that His2Av could be required for establishment of telomeric heterochromatin, a necessary step for telomeric piRNA cluster transcriptional activation.

We also report here, based on results from clonal analyses, that incomplete silencing likely involves an epigenetic lock mechanism. Incomplete silencing can be uncoupled into two steps. First, target repression, or lack thereof, needs to be established. We show that this step can occur very early, during embryogenesis, and that establishment of repression may depend on the amount of maternally inherited homologous piRNAs. It may also depend on the strength of the targeted promoter, or on the genomic location of the target; indeed, we previously showed that the level of incomplete silencing by a given telomeric silencer varies depending on the euchromatic target in adult ovaries (Josse *et al.* 2008). The second step is to “lock” the repression state: either repression is active (ON) or not (OFF). Indeed, since we observe that both the ON and the OFF states are maintained through PGC development, there seems to be no cumulative repressive effect of piRNAs over time during development, which would result in late establishment of repression. The data presented here thus suggest that the lock is already in place in late embryos. What could the molecular nature of this lock be? Does it involve an inability of telomeric silencers to produce piRNA precursor transcripts, thereby impeding target repression? Or, are targets repressed by PIWI–piRNA complexes epigenetically locked during development? Since our results indicate that HP1a is required to produce wild-type levels of telomeric piRNAs (Todeschini *et al.* 2010, Figure 5, C and F), it is tempting to propose that *P-1152* resides in a telomeric region where the presence of HP1a fluctuates, mimicking PEV phenomenon. By extension it is possible that, in some PGCs, HP1a is present at the level of the telomeric transgene such that enough piRNAs are produced to establish repression of the euchromatic target, whereas in other PGCs, the telomeric transgene is devoid of HP1a and produces fewer piRNAs, resulting in the nonrepression of the euchromatic target. Such a “piRNA-production variegation” phenomenon could explain the variegating repression phenotype we observed with the *P-1152* silencer. However, if the variegating repression phenotype depended on the presence and absence of piRNA production, then progeny derived from OFF germline cysts would not inherit transgenic piRNAs and should be devoid of silencing properties. Indeed, ≈50% of germline cysts are OFF, suggesting that ≈50% of the embryos should be devoid of silencing capabilities at the adult stage. This has never been observed during years of maintaining various *P-1152* stocks: 100% of the progeny at each generation show ≈50% (variegated) repression. This suggests that piRNA production capacity is not bimodal (ON or OFF) but rather that different thresholds of piRNAs are required for inducing piRNA-producing loci through generations (maternal piRNA inheritance) and for establishing euchromatic target repression. We propose that the lock might involve early established chromatin modifications of the euchromatic target itself, such as H3K9me3 and HP1a, which have been observed on many euchromatic TE insertions in correlation with piRNA-mediated silencing (Wang and Elgin 2011; Sienski *et al.* 2012, 2015; Le Thomas *et al.* 2013; Rozhkov *et al.* 2013; Pezic *et al.* 2014; Yu *et al.* 2015). The deposition of these marks should depend on the piRNA threshold that, in the case of *P-1152*, might depend on HP1a. What could be particularly interesting is the identification of specific chromatin factors that maintain the repression OFF state despite the presence of homologous piRNAs. Thus, the properties of variegating transgene repression in female germ cells reported here (*i.e.*, early establishment in the embryo, maintenance throughout germ cell development, and resetting at each generation) provide clues for future studies aimed at understanding establishment and maintenance of epigenetic regulation of TEs.

## Supplementary Material

Supplemental material is available online at www.g3journal.org/lookup/suppl/doi:10.1534/g3.116.037291/-/DC1.
