# Supplementary figures and images for: From Embryo to Adult: piRNA-Mediated Silencing throughout Germline Development in Drosophila
Source: G3 (Bethesda). 2016 Dec 7;7(2):505–16. doi: 10.1534/g3.116.037291 (PMC5295597; doi:10.1534/g3.116.037291)

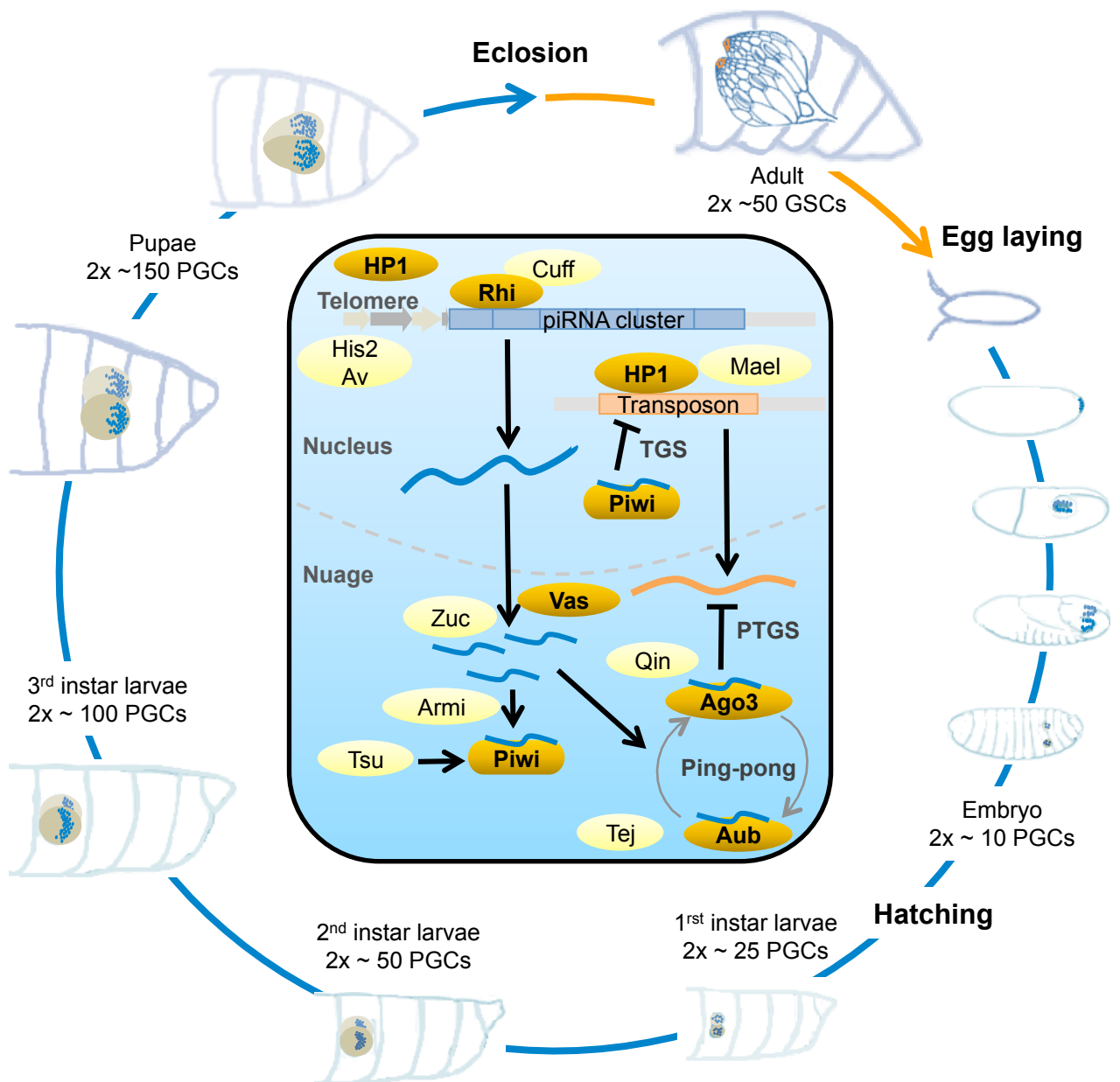

Supplement: Supplementary file 1 [file 505FigureS1.pdf]

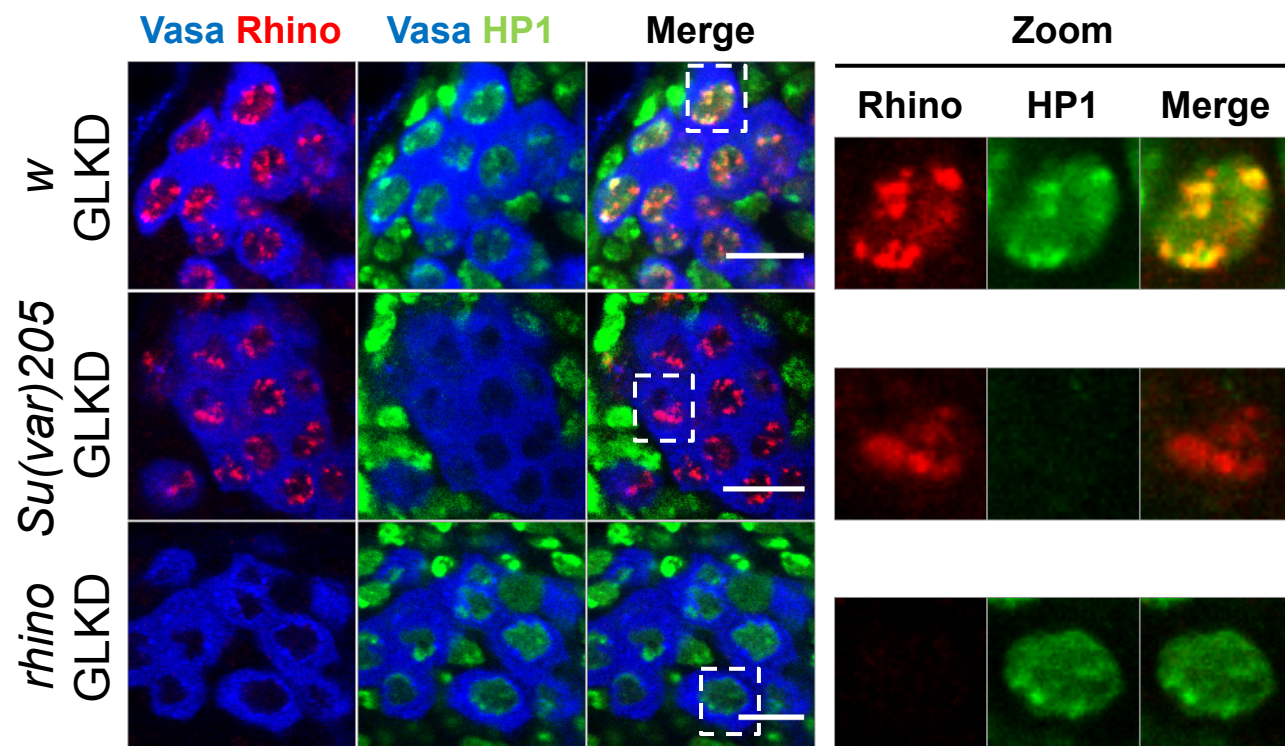

Supplement: Supplementary file 2 [file 505FigureS2.pdf]
