# Supplementary material for: From Embryo to Adult: piRNA-Mediated Silencing throughout Germline Development in Drosophila
Source: G3 (Bethesda). 2016 Dec 7;7(2):505–16. doi: 10.1534/g3.116.037291 (PMC5295597; doi:10.1534/g3.116.037291)
Supplement: Supplementary file 3 [file 505TableS1.pdf]

TABLE S1. Annotation of smRNA libraries.

Library	GRH113	GRH114	GRH115	GRH116	GRH141	GRH142	GRH154
Parental origin	w^{1118} x *RS3*	*RS3* x w^{1118}	w^{1118} x *RS3*	*RS3* x w^{1118}	*RS3, nosGAL4* x rhi^{GLKD}	*RS3, nosGAL4* x w^{GLKD}	*RS3, nosGAL4* x $Su(var)205^{GLKD}$
Code Figures 3 and 4	*RS3* paternal	*RS3* maternal	*RS3* paternal	*RS3* maternal	*rhino* GLKD	*w* GLKD	*Su(var)205* GLKD
Tissues	fat bodies + gonads	fat bodies + gonads	carcasses	carcasses	fat bodies + gonads	fat bodies + gonads	fat bodies + gonads
Dmel_matched (Flybase r5.49)	25576964	27619031	19072916	17531963	43157881	45540444	29067472
miRNAs	5119292	5259136	195788	190249	11081050	8758989	8374713
tRNAs	2022883	1009886	401397	510053	2585604	2780667	2908870
ncRNAs	810342	1143553	1635562	1537058	1045613	1063459	303859
miscRNAs (rRNA + snoRNAs)	14418738	16299122	13307916	11825247	24553017	26795350	14546798
Transposons	270387	194371	61404	78255	356280	404981	217087
Introns	527792	807826	746725	864245	882197	1278978	267667
Intergenics	564132	849103	336576	661979	821685	639535	383788
Transcripts	1600415	1908923	2361533	1835227	1316310	3352444	1625509
Remaining	242983	147111	26015	2965	516125	466041	439181
Effective depth (Dmel - tRNAs - miscRNAs)	9135343	10310023	5363603	5196663	16019260	15964427	11611804
Effective depth read per million (RPM) factor	0.109	0.097	0.186	0.192	0.062	0.063	0.086

Normalization factor (RPM factor x 5.2)	0.569	0.504	0.969	1.00	0.324	0.326	0.448
miRNA RPM factor	0.195	0.190	na	na	0.090	0.114	0.119

TABLE S1. Annotation of smRNA libraries. SmRNAs were prepared from either gonad-containing fat bodies or carcasses of female L3 larvae, as indicated ("Tissues" line). Genotypes are given in the "Parental origin" line (females x males). Each value for the different categories of sequences corresponds to the total number of sequence reads that matched either the *D. melanogaster* genome release 5.49 in each library ("Dmel Matched" line), or other annotated groups of sequences (from "miRNA" to "Remaining" lines). For comparisons, effective depth was calculated as the number of sequence reads that matched with the *D. melanogaster* genome but not with miscRNAs (rRNAs and snoRNAs) or tRNAs (Effective depth = Dmel matched - miscRNAs - tRNAs). Read per million factor is one million divided by effective depth. Library GRH116 which has the lowest effective depth, was taken as the reference to normalize the other libraries (Normalization factor = RPM factor x 5.2). miRNA RPM factor was also calculated for fat body libraries (miRNA amount was too low ($<10^6$) in carcass libraries to calculate RPM, na: not applicable).
