# Supplementary material for: From Embryo to Adult: piRNA-Mediated Silencing throughout Germline Development in Drosophila
Source: G3 (Bethesda). 2016 Dec 7;7(2):505–16. doi: 10.1534/g3.116.037291 (PMC5295597; doi:10.1534/g3.116.037291)
Supplement: Supplementary file 4 [file 505TableS2.pdf]

TABLE S2. *rhino* and *Su(var)205* GLKD effect on piRNA production in larval gonads.

	**GRH142**	**GRH141**	**GRH154**
	RS3, nosGAL4 x w^{GLKD}	*RS3, nosGAL4 x* rhi^{GLKD}	*RS3, nosGAL4 x Su(var)205*^{GLKD}
42AB (243,859 pb)	16.83 [30.67]	8.50 [12.29]	14.59 [20.23]
3R tip (11,331 pb)	20.82 [37.96]	4.24 [6.13]	5.66 [7.85]
RS3 transgene (6,051 pb)	11.27 [20.55]	1.82 [2.62]	2.25 [3.12]

TABLE S2. Reads per kilobase per million (RPKM) values were calculated for each genotype and for each locus as the number of reads that match the corresponding locus multiplied by the effective depth RPM factor (see Table S1) and divided by the size of the locus (in kb). In brackets, RPKM values calculated with miRNA RPM normalization (see Table S1) globally present higher RPKM values than those without this normalization and confirm results of the GLKD effects on telomeric piRNAs. This normalization also reveals a moderate effect of *Su(var)205* GLKD on *42AB* locus.
